# Cause of Mortality in Still-born Children

**Published:** 1848-02

**Authors:** 


9. *Cause of Mortality in Still-born Children.* (Ranking's Report in Abstract, January to July.)—The causes of deaths in still-births, with the means of preserving the infant's life, have been made the subject of an ingenious brochure by

Dr. Richard King, in which the author endeavors to show that the mortality arises from syncope, and not from asphyxia, as is commonly thought to be the case; and that the great danger to be dreaded in tedious or abnormal labors is not the compression of the cord so much talked of by accoucheurs, but its non-compression; so that the fœtal blood, as the placenta becomes detached, is, as it were, sucked up by that body, and the child is in fact thus, to all intents and purposes, rendered ex-sanguine. The treatment which the author suggests, as indicated by this theory, is the compression of the cord under certain circumstances, so as to prevent the possibility of that congestion of the placenta which he regards as the active cause of the death of the still-born infant. The arguments upon which the author founds his opinions are selected with judgment, and the work is altogether worthy of the best attention of the obstetrical practitioner.—*Ibid.*

